# Understanding the association between chromosomally integrated human herpesvirus 6 and HIV disease: a cross-sectional study

**DOI:** 10.12688/f1000research.2-269.v2

**Published:** 2017-06-01

**Authors:** Mundeep K. Kainth, Susan G. Fisher, Diana Fernandez, Amneris Luque, Caroline B. Hall, Anh Thi Hoang, Anisha Lashkari, Alexandra Peck, Lubaba Hasan, Mary T. Caserta

**Affiliations:** 1 Division of Pediatric Infectious Diseases, Department of Pediatrics, University of Rochester Medical Center, Rochester, NY, USA; 2 Department of Pharmacy, Temple University School of Medicine, Philadelphia, PA, USA; 3 Department of Public Health Sciences, University of Rochester Medical Center, Rochester, NY, USA; 4 Division of Infectious Diseases, Department of Medicine, University of Rochester Medical Center, Rochester, NY, USA

## Abstract

We conducted a cross-sectional investigation to identify evidence of a potential modifying effect of chromosomally integrated human herpes virus 6 (ciHHV-6) on human immunodeficiency virus (HIV) disease progression and/or severity. ciHHV-6 was identified by detecting HHV-6 DNA in hair follicle specimens of 439 subjects. There was no statistically significant relationship between the presence of ciHHV-6 and HIV disease progression to acquired immunodeficiency syndrome. However, after adjusting for use of antiretroviral therapy, all subjects with ciHHV-6 had low severity HIV disease; these findings were not statistically significant. A multi-center study with a larger sample size will be needed to more precisely determine if there is an association between ciHHV-6 and low HIV disease severity.

## Introduction

The relationship between chromosomally integrated human herpesvirus 6 (ciHHV-6) and the progression of human immunodeficiency virus (HIV) infection in humans has, to date, never been examined.

HHV-6 causes ubiquitous human infection with approximately 100% of the population seropositive by 3 years of age. The virus infects lymphocytes with a predominant tropism for CD4+ T cells ^1^. In individuals with ciHHV-6, the entire viral genome is present in every nucleated cell of the body and transmitted in a Mendelian fashion ^2, 3^. The presence of HHV-6 DNA in hair follicle DNA specimens is a marker for ciHHV-6 ^4^. Published data have demonstrated the prevalence of ciHHV-6 to be approximately 0.85% ^5^. If ciHHV-6 is associated with HIV infection, then this could uncover new possibilities for understanding HIV disease severity and progression.

Numerous studies have attempted to unravel the relationship between HHV-6 and HIV. Initially, the identification of HHV-6 (not ciHHV-6) DNA in 100% of saliva samples from HIV-infected adults with high CD4+ T-cell counts suggested a protective effect of HHV-6 on the progression of HIV ^6, 7^. HHV-6 produces a functional chemokine, U83A, which binds C-C chemokine receptor 5 (CCR5) and blocks HIV infection of susceptible cells *in vitro* providing a plausible mechanism for HHV-6 inhibition of HIV ^8^.

Alternatively, *in vitro* activation of the HIV long terminal repeat (LTR) sequences by HHV-6 has been described suggesting that HHV-6 may promote HIV disease progression ^9^. More recent data from animal models have linked infection with HHV-6 and the production of RANTES, the ligand for CCR5, with the acceleration of HIV disease via increased HIV virulence ^10, 11^. Clinical studies have found reactivation or persistence of HHV-6 antigen and IgM antibody in patients with progression from asymptomatic HIV to AIDS ^12^.

After acquiring HIV infection, approximately 80% of adults gradually develop low CD4+ T-cell counts over 5–10 years in the peripheral blood with a high HIV viral burden, and are otherwise referred to as ‘typical progressors’ (TP). A small proportion of individuals develop low CD4+ T-cell counts and high viral load within two years of initial infection; these individuals are identified as ‘rapid progressors’ (RP). The mechanisms surrounding these differences are unclear ^13, 14^. Additionally, despite prior investigations, there is also no clear understanding of the role of HHV-6 in the pathophysiology of HIV disease.

We conducted an exploratory investigation to estimate the prevalence of ciHHV-6 in an HIV-infected population and to determine if there is a potential modifying effect of ciHHV-6 on HIV disease progression or severity.

## Materials and methods

### Study design

This cross-sectional study included patients seen at the Infectious Diseases Clinic at Strong Memorial Hospital in Rochester, NY. The inclusion criterion limited the population to subjects with HIV infection over the age of 18. The exclusion criteria were lack of visible hair and inability to consent. Enrollers were available four days a week, 3–6 hours a day to approach eligible subjects.

### Consent and ethical considerations

Written informed consent for publication of their clinical details and/or clinical images was obtained from the patient/parent/guardian/ relative of the patient. This study protocol was reviewed and approved by the Research Subject Review Board at the University of Rochester (RSRB00032054).

### Data collection

Approximately one-half of the patients’ clinical data was obtained through the HIV Data Registry supported by the Developmental Center for AIDS Research (D-CFAR). The remainder of the patient data were obtained from electronic medical records with the use of a standardized chart abstraction tool (see data file) that included HIV diagnoses dates, date of AIDS defining illnesses, and viral load/CD4+ T cell counts. Demographic data such as race/ethnicity and sex/gender were provided by subject self identification and included in grant agency reports. Hair follicles were collected by enrollers by pulling 2–3 strands of scalp hair with gloved hands.

Based on established standards, the definition of RP HIV included patients with CD4+ T cell counts of less than 200 or an AIDS-defining illness within 2 years of diagnosis of HIV infection. Subjects who had confirmed HIV infection did not meet criteria for RP were placed in a TP group. Diagnosis of HIV infection was identified by a positive ELISA/Western blot test or indicated by a provider in chart documentation based on patient history. Testing for HIV during the study period was performed by antibody testing (Vitros ECI) at Strong Memorial Hospital. Confirmation by Western blot (INNO-LIA HIV I/II Score) was performed at the Mayo Clinic in Rochester, MN.

We also compared HIV disease severity in ciHHV-6 positive and negative patients because initial disease progression from HIV to AIDS may not necessarily correlate with current disease severity in subjects on antiretroviral treatment (ART). Subjects were considered to have severe disease if CD4+ T cell counts were less than 200 cells/uL and viral load was greater than 10,000 copies/mL. If CD4+ T cell counts were greater than 200 cells/uL, and viral load was less than 10,000 copies/mL, subjects were considered to have non-severe disease. All subjects with any remaining combination of viral load and CD4+ T cell counts were considered to have ‘moderately’ severe disease.

### HHV-6 testing

Hair samples were chosen to identify HIV positive subjects with ciHHV-6 and to exclude subjects with acquired HHV-6 infection. Hair follicle samples were digested with proteinase K followed by DNA extraction using QIAamp DNA mini kit (Valencia, CA). DNA samples were tested by a nested qualitative PCR amplifying the HHV-6 U38 DNA polymerase gene as previously described ^15^. In order to verify the presence of ciHHV-6, qualitative PCR was repeated on all of the positive samples followed by quantitative real time PCR for the HHV-6 U4 gene as described by Zhen *et al.* ^16^ with modifications developed in our laboratory ^17^.

### Data analysis

All data was analyzed with SAS (v9.2). Prevalence rates of ciHHV-6 were compared between the entire cohort of HIV-infected subjects and the prevalence of ciHHV-6 in the general population ^5^. Next, the prevalence of ciHHV-6 was compared between RP and TP subjects to determine if there was a significant difference of ciHHV-6 between these two groups. We also determined whether the presence of ciHHV-6 was associated with markers of HIV disease severity ascertained at the time of enrollment.

Student’s t-test was used to compare age and the chi-square test for gender, ethnicity, and race. An analysis of subjects positive for ciHHV-6 and markers of HIV disease progression was performed comparing prevalence rates between the RP and TP groups and the three severity groups using Fisher’s exact test. An association was considered to be statistically significant at p<0.05.

## Results

### Cohort

The clinic population available for this study included 1035 HIV-infected patients, 714 men and 321 women between 18 and 79 years of age (African American – 418 (40%); White – 453 (44%); Hispanic – 155 (7%); other – 9 (<0.01%)). Approximately115 patients per week were invited to join this study during routine clinic visits from October 2010 to April 2012 and 463 subjects were enrolled. From this group, 1 subject withdrew and 9 were erroneously enrolled twice. 4 subjects with unobtainable hair follicle DNA were excluded. Clinical data on 215 subjects were obtained from the University of Rochester D-CFAR database. For the remaining 248 subjects, electronic medical records were reviewed. When discrepancies between HIV and AIDS diagnosis dates occurred, the earliest date identified on the chart was selected. HIV diagnosis date was unavailable for 10 subjects, with a final number of 439 subjects with complete data available for analysis.

Approximately 1/3 of the total cohort of subjects identified themselves as female, 2/3 male, and less than 1% were transgender. Reported racial distributions for enrolled subjects were: White 205 (47%), Black-African American 171 (39%), White-Black 5 (1%), unknown or other 54 (12.3%), Asian 1, and Hawaiian Pacific-Islander 1 ( Table 1). Fifty-eight (13.2%) subjects reported their ethnicity as Hispanic. Age ranged from 18 to 74 years with a mean age of 46.7 years and a median of 48 years.

**Table 1.** Demographic data by presence or absence of CI-HHV 6 ^a^ detected by quantitative PCR.

	Positive		Negative		*Total*	
**Gender**	**n**	**%**	**n**	**%**	**n**	**%**
*Male*	2	0.5	287	65.4	289	65.8
*Female*	2	0.5	145	33.0	147	33.5
*Transgender*	0	0	3	0.7	3	0.7
**Race**	**n**	**%**	**n**	**%**	**n**	**%**
*Asian*	0	0	1	0.2	1	0.2
*Black/AA* ^b^	2	0.5	169	38.5	171	39.0
*Other*	0	0	8	1.8	8	1.8
*Unknown*	0	0	48	11.0	48	11.0
*White*	2	0.5	203	46.2	205	46.7
*White-Black*	0	0	5	1.1	5	1.1
*White-Haw/PI* ^c^	0	0	1	0.2	1	0.2
**Ethnicity**	**n**	**%**	**n**	**%**	**n**	**%**
*Hispanic*	0	0	58	13.2	58	13.2
*Non-Hispanic*	4	0.9	347	79.0	351	80.0
*Unknown*	0	0	30	6.8	30	6.8

**^a^**CI-HHV6 (Chromosomally Integrated HHV6)

**^b^**AA (African-American)

**^c^**Haw/PI (Hawaiian/Pacific-Islander)

### ciHHV-6 results

Eight ciHHV-6 positive samples out of a total of 439 hair follicle samples were identified by initial qualitative PCR testing. Upon re-testing, only three samples were positive. Real-time quantitative PCR analysis verified 4 positive samples ( Table 2). Due to the higher specificity of the assay, the quantitative PCR data were used in the remaining study analyses.

**Table 2.** Quantitative PCR results from hair follicle specimens for ciHHV-6 ^a^ positive subjects.

Patient ID number	Log _10_ gec per μg of DNA ^b^
165	7.93
254	5.61
258	8.05
369	7.87

**^a^**ciHHV-6 (chromosomally integrated human herpes virus 6)

**^b^**Values >5 Log _10_ genome equivalent copies (gec) per μg of DNA are considered to be positive for high viral loads of HHV-6 DNA indicating chromosomal integration

### Prevalence of ciHHV-6 in study population

The calculated prevalence estimate of ciHHV-6 in the cohort was 0.91% with a 95% CI of [0.37–2.31%]. Of the four positive ciHHV-6 subjects, 2 were female and 2 were male; 2 were white and 2 were black.

### HIV disease progression and ciHHV-6

Of 340 (77%) TP subjects, 3 (0.68%) were positive for ciHHV-6. Ninety-nine (23%) subjects were classified as having RP HIV disease based on the time to AIDS diagnosis after the date of HIV infection. Out of these 99 subjects, one (0.23%) was positive for ciHHV-6. Due to the low ciHHV-6 prevalence rate in the study population, the difference between ciHHV-6 among RP and TP is not statistically significant (p<0.42).

### HIV disease severity and ciHHV-6

Patients were categorized into three severity groups based on their CD4+ T cell count and HIV viral loads as described above. In order to control for antiretroviral therapy (ART) use, 9 individuals who were ART naïve were excluded. 332 subjects (84%) had non-severe disease, 42 (11%) had moderately severe disease, and 19 (5%) had severe disease. All of the subjects with ciHHV-6 had non-severe HIV disease. Given the small numbers, this finding did not reach statistical significance (p<0.51).

Dataset 1: Data on ciHHV-6 presence and HIV disease progression in 439 patients at the Infectious Diseases Clinic at Strong Memorial Hospital in Rochester, NY PID: Subject identification; Last_VL: Subject’s most recent viral load; Last_CD4: Subject’s most recent CD4+ T-cell count;Last_CD4_%: Subject’s most recent CD4+ T-cell count percent; Time_to_progression: time (in years) between HIV diagnosis and AIDS diagnosis; Rapid Progressor: Less than two years to between HIV and AIDS diagnosis; ART Naïve: If subject is naïve to antiretroviral therapy – ‘yes’; CIHHV6Run2: Subject positive for qualitative PCR results – ‘1’; QuantPCR: Subject positive for quantitative PCR results – ‘1’. Click here for additional data file. Copyright: © 2017 Kainth MK et al. 2017 Data associated with the article are available under the terms of the Creative Commons Zero "No rights reserved" data waiver (CC0 1.0 Public domain dedication).

## Discussion

This pilot study is the first to identify the presence of ciHHV-6 in an HIV-infected population. Because ciHHV-6 is inherited in a Mendelian fashion, our data confirmed that the prevalence rate of ciHHV-6 in our total HIV-infected cohort was similar to the published prevalence rate in otherwise healthy populations. We hypothesized that ciHHV-6 would be associated with rapid HIV disease progression or markers of disease severity. However, we did not identify a significant association between HIV disease progression and ciHHV-6 status. All four subjects with ciHHV-6 had non-severe disease; yet this was also not significant due to the small numbers of individuals with ciHHV-6 identified.

The strengths of the study include that a large number of HIV-infected subjects were available for study and the ease of hair sample collection. The testing was appealing to subjects and allowed efficient sample storage. The major limitation of this study was the low prevalence of ciHHV-6 in the HIV-infected cohort. Additionally, we were not always able to identify dates for HIV infection and AIDS diagnosis to establish clinical disease progression. We attempted to overcome differential misclassification by evaluating CD4+ T cell counts and viral loads at the time of sample collection to establish a subject’s HIV disease severity. This last method revealed that all 4 subjects with ciHHV-6 were in the low disease severity group, but due to the low ciHHV-6 prevalence, we were unable to conduct further statistical analyses.

While earlier *in vitro* studies have suggested a protective effect of HHV-6 infection on HIV disease, our study is the first clinical investigation to identify a possible protective effect of ciHHV-6 on HIV disease severity. However, due to a lack of statistical significance of the association between ciHHV-6 and HIV disease severity, more data will need to be collected to assess this relationship.
