# Supplementary material for: Understanding the association between chromosomally integrated human herpesvirus 6 and HIV disease: a cross-sectional study
Source: F1000Res. 2017 Jun 1;2:269. Originally published 2013 Dec 9. [Version 2] doi: 10.12688/f1000research.2-269.v2 (PMC3892924; doi:10.12688/f1000research.2-269.v2)
Supplement: Peer review history

# Peer review: Referee response for version 1

**DOI:** 10.5256/f1000research.2563.r2695

**Published:** 2014-01-09

**Authors:** David Camerini

**Affiliations:** 1 Department of Molecular Biology and Biochemistry, University of California, Irvine, CA, USA

This interesting, well-conducted study investigated the relationship between chromosomally integrated human herpes virus six (ciHHV-6) on HIV disease severity and progression. Individuals with ciHHV-6 constitute just under 1% of the general population and a similar proportion of the 439 HIV positive individuals analyzed in this study. Due to the small sample size, only 4 ciHHV-1, HIV+ individuals were found and no significant conclusions could be drawn regarding any association with HIV disease. Nevertheless, this study is thought provoking and may provide impetus for a larger study that could shed light on this potential viral interaction *in vivo*.

I have read this submission. I believe that I have an appropriate level of expertise to confirm that it is of an acceptable scientific standard.

# Peer review: Referee response for version 1

**DOI:** 10.5256/f1000research.2563.r3026

**Published:** 2014-01-07

**Authors:** Mario Clerici

**Affiliations:** 1 Department of Biomedical Sciences and Technology, University of Milano, Milano, 20157, Italy

Because of the very low ciHHV-6 positivity in the analyzed HIV-infected population, the author's suggestion that the presence of ciHHV-6 associates with HIV disease severity seems to be too strong and not (yet) justified.

In particular, the abstract is misleading: the authors should add the number of ciHHV-6 positive subjects, and delete/modify the sentence about the severity of disease and ciHHV-6.

I have read this submission. I believe that I have an appropriate level of expertise to confirm that it is of an acceptable scientific standard, however I have significant reservations, as outlined above.

# Peer review: Referee response for version 1

**DOI:** 10.5256/f1000research.2563.r2694

**Published:** 2014-01-07

**Authors:** Susana N. Asin

**Affiliations:** 1 Department of Microbiology and Immunology, Dartmouth Medical School, Hanover, NH, USA

This study addresses an interesting question: the potential impact of chromosomally integrated Human Herpes Virus 6 (HHV6) on HIV-1 disease progression. The rationale underlying the proposed hypothesis is weak. The authors do not suggest potential mechanisms underlying their hypothesis, which may provide a more provoking discussion. It is appropriate to anticipate that immunosuppression will lead to HHV6 reactivation. Since HHV6 shares tropism for CD4 ^+ ^T cells with HIV-1, HHV6 could accelerate disease progression by impairing the immune response to HIV-1. Based on observation demonstrating that HHV6 replication accelerates progression to AIDS in macaques, I am curious to understand why the authors hypothesize that chromosomally integrated HHV6 could impact HIV-1 disease progression specially without evaluating HHV6 replication/reactivation.

It would have been appropriate to define how the authors evaluated chromosomally integrated DNA. Are the primers used specific for HHV6 or do these primers cross react with other HHV family members? Only describing the target gene will allow the reader to define the potential confounding effects of additional endogenous herpes viruses.

The manuscript is well written and the authors do not draw overambitious conclusions. The fact that only one individual in the rapid progression group had ciHHV6 support the author’s conclusion to conduct a multi-center study with a larger sample size. Based on higher HHV6 prevalence rates described in another groups of HIV-1 infected individuals I would be curious to know whether the authors evaluated HHV6 antibodies in this group and how do they explained that, despite high prevalence, the percentage of integrated HHV6 DNA is so low. The pathogenesis of HHV6 in HIV-1 infected individuals should be further investigated.

I have read this submission. I believe that I have an appropriate level of expertise to confirm that it is of an acceptable scientific standard.
